# Crystal structure and Hirshfeld surface analysis of dimethyl 5-[2-(2,4,6-trioxo-1,3-diazinan-5-yl­idene)hydrazin-1-yl]benzene-1,3-di­carboxyl­ate 0.224-hydrate

**DOI:** 10.1107/S2056989021006563

**Published:** 2021-06-30

**Authors:** Zeliha Atioğlu, Mehmet Akkurt, Gunay Z. Mammadova, Fatali E. Huseynov, Sevinj R. Hajiyeva, Nazim T. Shamilov, Ajaya Bhattarai

**Affiliations:** a Department of Aircraft Electrics and Electronics, School of Applied Sciences, Cappadocia University, Mustafapaşa, 50420 Ürgüp, Nevşehir, Turkey; b Department of Physics, Faculty of Sciences, Erciyes University, 38039 Kayseri, Turkey; c Department of Chemistry, Baku State University, Z. Khalilov str. 23, AZ, 1148 Baku, Azerbaijan; d Department of Ecology and Soil Sciences, Baku State University, Z. Khalilov str. 23, AZ, 1148 Baku, Azerbaijan; e Department of Chemistry, M.M.A.M.C (Tribhuvan University) Biratnagar, Nepal

**Keywords:** crystal structure, 1,3-diazinane ring, hydrogen bonds, Hirshfeld surface analysis

## Abstract

In the crystal, the whole mol­ecule of the title compound, C_14_H_12_N_4_O_7_·0.224H_2_O, is nearly planar with a maximum deviation from the least-squares plane of 0.352 (1) Å. The mol­ecular conformation is stabilized by an intra­molecular N—H⋯O hydrogen bond, generating an *S*(6) ring motif. In the crystal, mol­ecules are linked by centrosymmetric pairs of N—H⋯O hydrogen bonds, forming ribbons along the *c-*axis direction. These ribbons connected by van der Waals contacts, forming sheets parallel to the *ac* plane. There are also inter­molecular van der Waals contacts and and C—H⋯π inter­actions between the sheets. A Hirshfeld surface analysis indicates that the most prevalent inter­actions are O⋯H/H⋯O (41.2%), H⋯H (19.2%), C⋯H/H⋯C (12.2%) and C⋯O/ O⋯C (8.4%).

## Abstract 2

In the crystal, mol­ecules are linked by pairs of N—H⋯O hydrogen bonds into ribbons along the *c-*axis direction. The layered crystal packing is further consolidated by van der Waals and C—H⋯π inter­actions.

## Chemical context   

Aryl­hydrazones, besides their biological significance (Viswanathan *et al.*, 2019 ▸), can also be used as precursors in the synthesis of coordination compounds (Gurbanov *et al.*, 2017 ▸, 2018*a* ▸,*b* ▸; Ma *et al.*, 2017*a* ▸,*b* ▸) and as building blocks in the construction of supra­molecular structures owing to their hydrogen-bond donor and acceptor capabilities (Mahmoudi *et al.*, 2016 ▸, 2017*a* ▸,*b* ▸,*c* ▸, 2018*a* ▸,*b* ▸; 2019 ▸). All the reported hydrazone ligands are stabilized in the hydrazone form by intra­molecular resonance-assisted hydrogen bonding (RAHB) between the hydrazone =N—NH— fragment and the carbonyl group, giving a six-membered ring (Gurbanov *et al.*, 2020*a* ▸; Kopylovich *et al.*, 2011*a* ▸,*b* ▸; Mizar *et al.*, 2012 ▸). The use of multifunctional ligands in coordination chemistry is a common way to increase the water solubility of metal complexes, which is important for catalytic applications in aqueous medium (Ma *et al.*, 2020 ▸, 2021 ▸; Mahmudov *et al.*, 2013 ▸; Sutradhar *et al.*, 2015 ▸, 2016 ▸). Moreover, non-covalent inter­actions such as hydrogen, halogen and chalcogen bonds as well as π-inter­actions or their cooperation are able to contribute to synthesis and catalysis and improve the properties of materials (Gurbanov *et al.*, 2020*b* ▸; Karmakar *et al.*, 2017 ▸; Khalilov *et al.*, 2018*a* ▸,*b* ▸; Mac Leod *et al.*, 2012 ▸; Shikhaliyev *et al.*, 2019 ▸; Shixaliyev *et al.*, 2014 ▸). For that, the main skeleton of the hydrazone ligand should be decorated by non-covalent bond donor centre(s). In a continuation of our work in this area, we have prepared a new hydrazone ligand, dimethyl 5-{2-[2,4,6-trioxo­tetra­hydro­pyrimidin-5(2*H*)-yl­id­ene] hydrazine­yl}isophthalate, which provides the centres for coordination and inter­molecular non-covalent inter­actions.
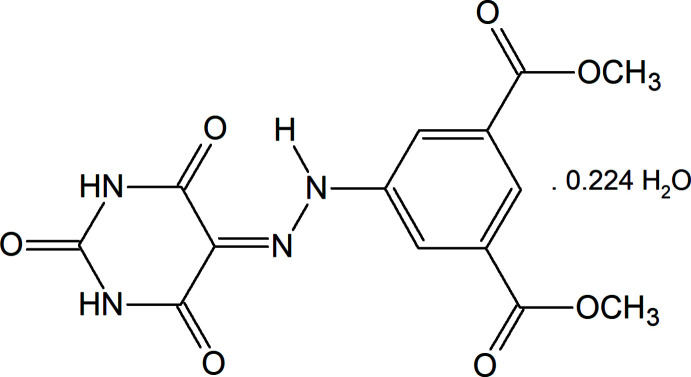


## Structural commentary   

**Figure 1:**
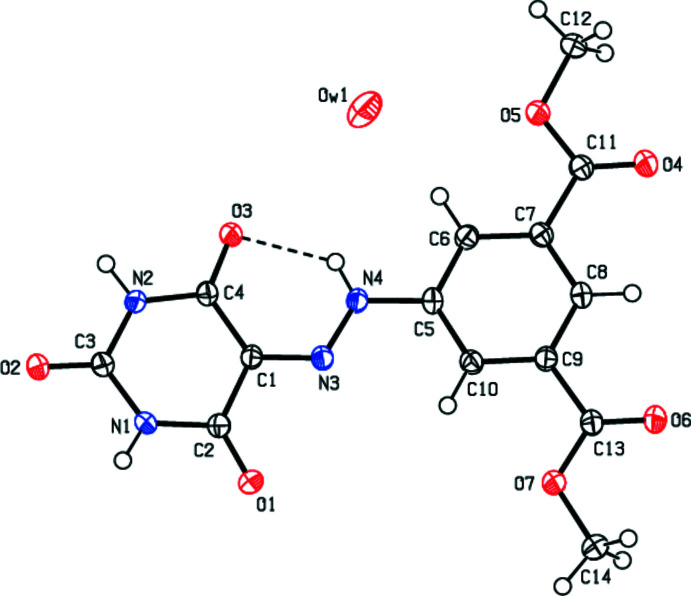
The mol­ecular structure of the title compound, showing the atom labelling and displacement ellipsoids drawn at the 50% probability level.

The asymmetric unit of the title structure contains one title mol­ecule and a water mol­ecule, which partially occupies a small cavity with an occupancy factor of 0.224 (5). The title mol­ecule (Fig. 1 ▸) is nearly planar with the largest deviation from the least-squares plane being 0.352 (1) Å for the methyl­carboxyl­ate atom O6. The 1,3-diazinane ring makes a dihedral angle of 9.96 (5)° with the benzene ring. The planar mol­ecular conformation is stabilized by an intra­molecular N—H⋯O contact (Table 1 ▸), generating an *S*(6) ring motif (Bernstein *et al.*, 1995 ▸).

## Supra­molecular features   

**Figure 2:**
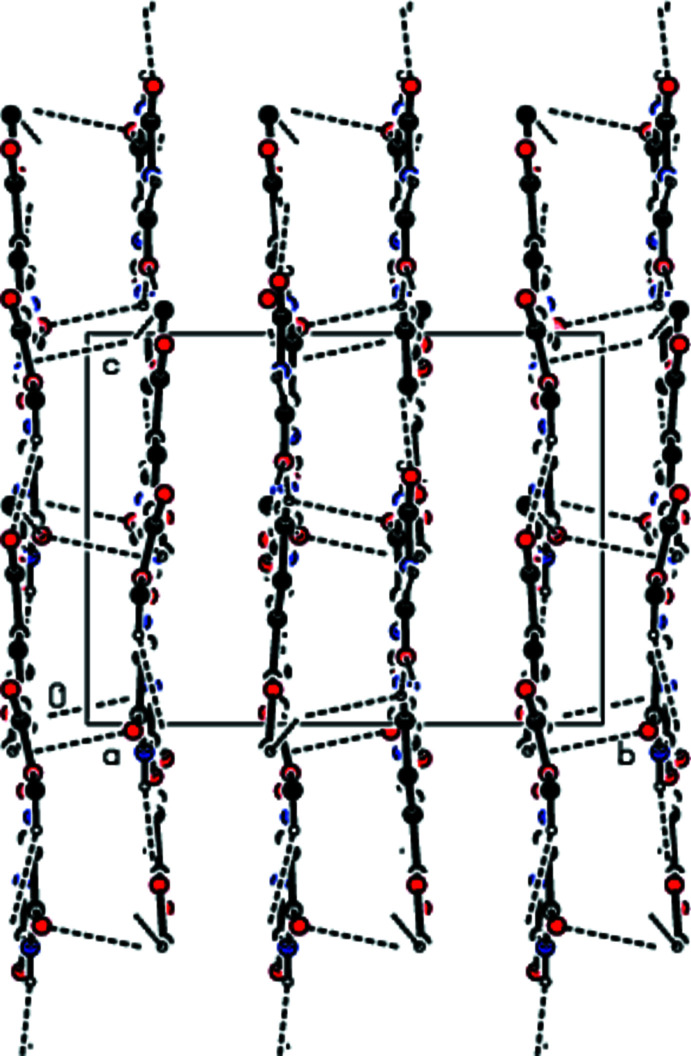
A view down the *a* axis showing the inter­molecular contacts forming the layered structure.

In the crystal, the mol­ecules are linked by pairs of N—H⋯O hydrogen bonds into ribbons along the *c*-axis direction (Table 1 ▸). These ribbons are connected by van der Waals inter­actions, forming sheets parallel to the *ac* plane. There are also other van der Waals contacts and C—H⋯π inter­actions between the sheets (Table 2 ▸), consolidating the crystal packing (Figs. 2 ▸–4 ▸ ▸).

## Hirshfeld surface analysis   

**Figure 5:**
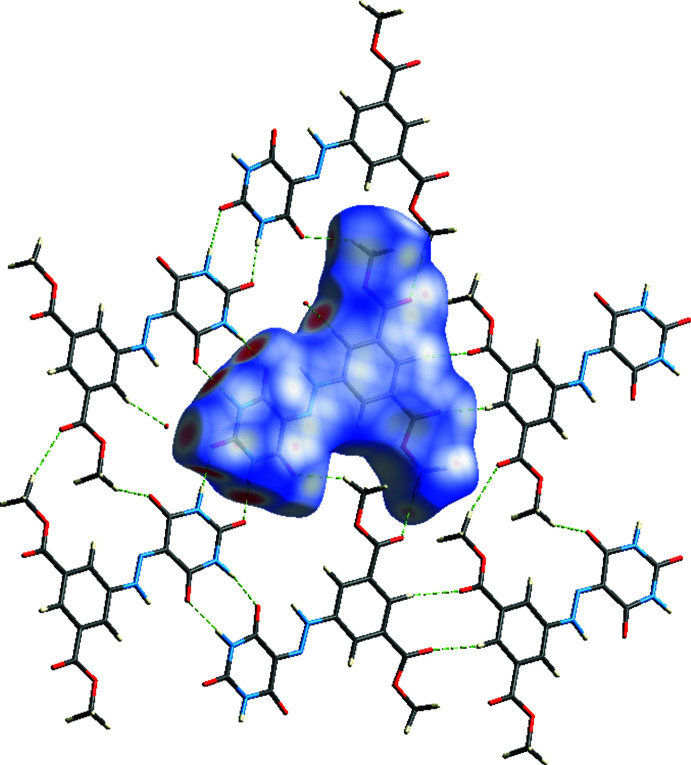
A view of the Hirshfeld surface mapped over *d*_norm_, with inter­actions to neighbouring mol­ecules shown as green dashed lines.

**Figure 6:**
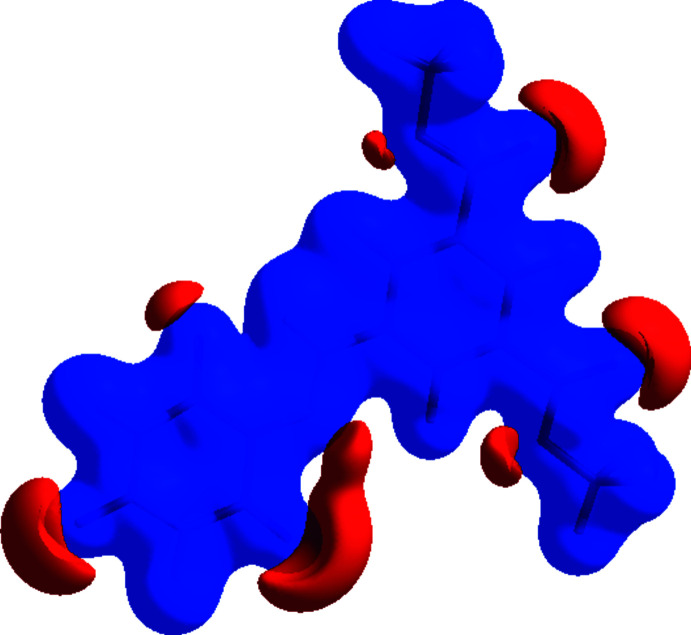
The Hirshfeld surface of the title compound plotted over electrostatic potential energy in the range from −0.0500 to 0.0500 a.u. using the STO-3G basis set at the Hartree–Fock level of theory. Hydrogen-bond donors and acceptors are shown as blue and red regions around the atoms, corresponding to positive and negative potentials, respectively.

**Figure 7:**
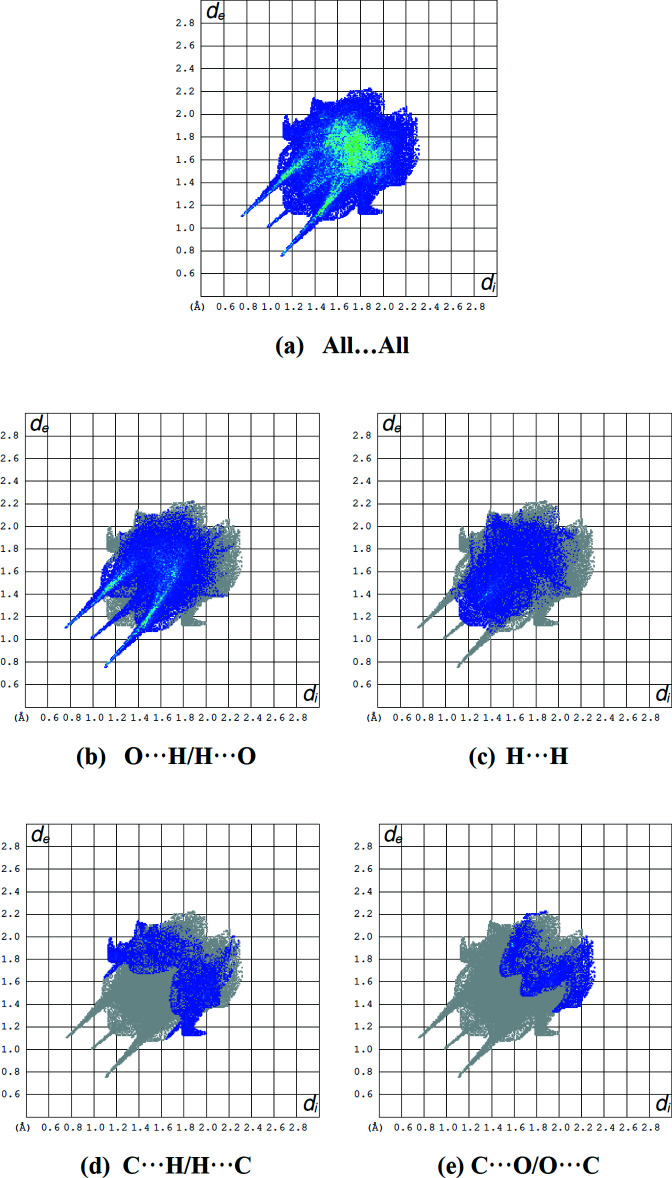
The two-dimensional fingerprint plots of the title compound, showing (*a*) all inter­actions, and delineated into (*b*) O⋯H/H⋯O, (*c*) H⋯H, (*d*) C⋯H/H⋯C and (*e*) C⋯O/O⋯C, inter­actions [*d*_e_ and *d*_i_ represent the distances from a point on the Hirshfeld surface to the nearest atoms outside (external) and inside (inter­nal) the surface, respectively].

The Hirshfeld surface for the title mol­ecule was performed and its associated two-dimensional fingerprint plots were prepared using *Crystal Explorer 17* (Turner *et al.*, 2017 ▸) to further investigate the inter­molecular inter­actions in the title structure. The oxygen atom of the water mol­ecule with partial occupancy was not taken into account. The Hirshfeld surface mapped over *d*_norm_ with corresponding colours representing inter­molecular inter­actions is shown in Fig. 5 ▸. The red spots on the surface correspond to the N—H⋯O and C—H⋯O inter­actions (Tables 1 ▸ and 2 ▸). The Hirshfeld surface mapped over electrostatic potential (Spackman *et al.*, 2009 ▸) is shown in Fig. 6 ▸. The blue regions indicate positive electrostatic potential (hydrogen-bond donors), while the red regions indicate negative electrostatic potential (hydrogen-bond acceptors). The two-dimensional fingerprint plots (McKinnon *et al.*, 2007 ▸) are shown in Fig. 7 ▸. O⋯H/H.·O contacts make the largest contribution (41.2%; Fig. 7 ▸ *b*) to the Hirshfeld surface. The other large contributions to the Hirshfeld surface are from H⋯H (19.2%; Fig. 7 ▸ *c*), C⋯H/H⋯C (12.2%; Fig. 7 ▸ *d*) and C⋯O/O⋯C (8.4%; Fig. 7 ▸ *e*) inter­actions. All contributions to the Hirshfeld surface are listed in Table 3 ▸. These inter­actions play a crucial role in the overall cohesion of the crystal packing.

## Database survey   

A search of Cambridge Crystallographic Database (CSD, version 5.40, update of September 2019; Groom *et al.*, 2016 ▸) was undertaken for structures containing the 5-(2-methyl­hydrazinyl­idene)-1,3-diazinane moiety. The first three structures are free bases are: 2-{2-[(1*H*-imidazol-5-yl)methyl­idene]-1-methyl­hydrazin­yl}pyridine (QUGVEW; Bocian *et al.*, 2020 ▸), 2-{2-[(1*H*-imidazol-2-yl)methyl­idene]-1-methyl­hydrazin­yl}-1*H*-benzimidazole monohydrate (QUGVIA; Bocian *et al.*, 2020 ▸) and 2-{1-methyl-2-[(1-methyl-1*H*-imidazol-2-yl)methyl­idene]hydrazin­yl}-1*H*-benzimidazole hydrate unknown solvate (QUGVOG; Bocian *et al.*, 2020 ▸). The other two are triflate salts are: 5-{[2-(1*H*-benzimidazol-2-yl)-2-methyl­hydrazinyl­idene]meth­yl}-1*H*-imidazol-3-ium tri­fluoro­meth­ane­sulfonate monohydrate (QUGVUM; Bocian *et al.*, 2020 ▸) and (2-{2-[(1*H*-imidazol-3-ium-2-yl)methyl­ene]-1-methyl­hydrazine­yl}pyridin-1-ium) bis­(tri­fluoro­methane­sulfonate) (QUGWAT; Bocian *et al.*, 2020 ▸).

In the structures of QUGVEW, QUGVIA, QUGVOG, QUGVUM and QUGWAT, the most important contribution to the stabilization of the crystal packing is provided by π–π inter­actions, especially between cations in the structures of salts, while the characteristics of the crystal architecture are influenced by directional inter­actions, especially relatively strong hydrogen bonds. In one of the structures (QUGWAT), an inter­esting example of a non-typical F⋯O inter­action was found whose length, 2.859 (2) Å, is one of the shortest ever reported.

## Synthesis and crystallization   

**Diazo­tization:** 2.09 g (10 mmol) of dimethyl 5-amino­isophthalate were dissolved in 50 mL of water, the solution was cooled on an ice bath to 273 K and 0.69 g (10 mmol) of NaNO_2_ were added; 2.00 mL of HCl were then added in 0.5 mL portions over 1 h. The temperature of the mixture should not exceed 278 K.

**Azocoupling:** NaOH (0.40 g, 10 mmol) was added to a mixture of 10 mmol (1.28 g) of barbituric acid with 25.00 mL of water. The solution was cooled on an ice bath and a suspension of 3,5-*bis*(meth­oxy­carbon­yl)benzene­diazo­nium chloride, prepared according to the procedure described above, was added in two equal portions under vigorous stirring for 1 h. The formed precipitate of the title compound was filtered off, recrystallized from methanol and dried in air. Crystals suitable for X-ray analysis were obtained by slow evaporation of an ethanol solution.

**The title compound:** Yield, 68% (based on barbituric acid), yellow powder soluble in DMSO, methanol, ethanol and DMF. Analysis calculated for C_14_H_12_N_4_O_7_ (*M*_r_ = 348.27): C, 48.28; H, 3.47; N, 16.09; found: C, 48.25 H, 3.41; N, 16.03%. ESI–MS: *m*/*z*: 349.2 [*M*_r_ + H]^+^. IR (KBr): 3160, 3090 and 2846 *ν*(NH), 1745 and 1663 *ν*(C=O), 1610 *ν*(C=O⋯H) cm^−1. 1^H NMR (300.130 MHz) in DMSO-*d*_6_, inter­nal TMS, *δ* (ppm): 8.20–8.36 (3H, Ar—H), 11.32 (*s*, 1H, N—H), 11.54 (*s*, 1H, N—H), 14.08 (*s*, 1H, N—H). ^13^C{^1^H} NMR (75.468 MHz, DMSO-*d*_6_). *δ*: 55.6 (2OCH_3_), 119.54 (2Ar—H), 121.8 (Ar-H), 127.4 (2C—COOCH_3_), 133.25 (C=N), 142.87 (C—NHN=), 150.24 (C=O), 160.32 (C=O), 161.90 (C=O⋯H) and 166.56 (2COOCH_3_).

## Refinement details   

Crystal data, data collection and structure refinement details are summarized in Table 4 ▸. The H atoms of the NH groups were located by difference Fourier synthesis and their coord­inates were fixed. All C-bound H atoms were positioned geometrically and refined using a riding model, with C—H = 0.95 and 0.98 Å, and with *U*_iso_(H) = 1.2 or 1.5*U*_eq_(C). There is a small cavity in the crystal, which is only partially occupied by a water mol­ecule, with an occupancy of 0.224 (5), and its hydrogen atoms could not be located.

## Supplementary Material

Crystal structure: contains datablock(s) I. DOI: 10.1107/S2056989021006563/yk2153sup1.cif

Structure factors: contains datablock(s) I. DOI: 10.1107/S2056989021006563/yk2153Isup2.hkl

Supporting information file. DOI: 10.1107/S2056989021006563/yk2153Isup3.cml

CCDC reference: 2091530

Additional supporting information:  crystallographic information; 3D view; checkCIF report

## Figures and Tables

**Figure 3:**
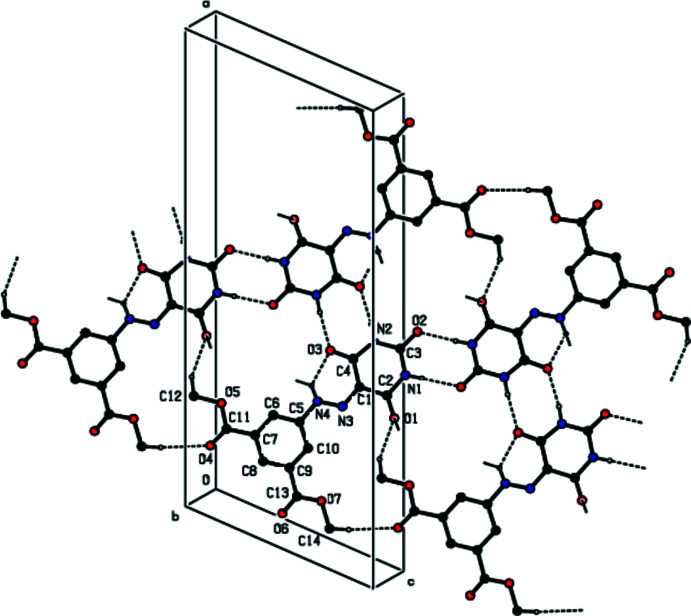
A view of inter­molecular hydrogen bonds forming the ribbons along the *c*-axis direction.

**Figure 4:**
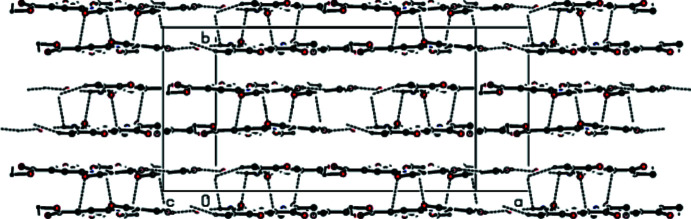
A view of the projection on the *ab* plane showing the contacts between layers.

**Table 1:** Hydrogen-bond geometry (Å, °) *Cg*2 is the centroid of the C5–C10 benzene ring.

*D*—H⋯*A*	*D*—H	H⋯*A*	*D*⋯*A*	*D*—H⋯*A*
N1—H1*N*⋯O2^i^	0.86	2.03	2.8800 (13)	174
N2—H2*N*⋯O3^ii^	0.90	2.01	2.8931 (15)	168
N4—H4*N*⋯O3	0.86	2.02	2.6571 (15)	131
C6—H6⋯Ow1	0.95	2.14	3.061 (6)	163
C12—H12*B*⋯O1^iii^	0.98	2.39	3.2743 (17)	149
C14—H14*B*⋯O4^iv^	0.98	2.53	3.4754 (16)	163
C12—H12*C*⋯*Cg*2^v^	0.98	2.73	3.4717 (15)	133

Symmetry codes: (i) -x+1, y, -z+{\script{5\over 2}}; (ii) -x+1, y, -z+{\script{3\over 2}}; (iii) x, y, z-1; (iv) x, y, z+1; (v) x, -y+1, z-{\script{1\over 2}}.

**Table 2:** Summary of short inter­atomic contacts (Å) in the title compound

Contact	Distance	Symmetry operation
O1⋯*Ow1	3.129	*x*, *y*, 1 + *z*
O1⋯H12*B*	2.39	*x*, *y*, 1 + *z*
O1⋯H4*N*	2.59	*x*, 1 − *y*, {1\over 2} + *z*
H12*A*⋯O1	2.67	{1\over 2} − *x*, {1\over 2} − *y*, 1 − *z*
H1*N*⋯O2	2.03	1 − *x*, *y*, {5\over 2} − *z*
O2⋯*Ow1	2.662	1 − *x*, *y*, {3\over 2} − *z*
N2⋯O2	3.226	1 − *x*, 1 − *y*, 2 − *z*
O2⋯H14*C*	2.64	{1\over 2} + *x*, {1\over 2} − *y*, {1\over 2} + *z*
H2*N*⋯O3	2.01	1 − *x*, *y*, {3\over 2} − *z*
H4*N*⋯O1	2.59	*x*, 1 − *y*, − {1\over 2} + *z*
H12*B*⋯O1	2.39	*x*, *y*, − 1 + *z*
H8⋯O6	2.66	−*x*, *y*, {1\over 2} − *z*
H14*A*⋯O6	2.67	−*x*, 1 − *y*, 1 − *z*
H14*C*⋯O2	2.64	−{1\over 2} + *x*, {1\over 2} − *y*, − {1\over 2} + *z*
C1⋯*Ow1	3.297	*x*, 1 − *y*, {1\over 2} + *z*
H6⋯*Ow1	2.14	*x*, *y*, *z*
H12*B*⋯C12	3.10	{1\over 2} − *x*, {1\over 2} − *y*, −*z*
H14*B*⋯C14	2.93	−*x*, *y*, {3\over 2} − *z*
H12*A*⋯*Ow1	2.70	{1\over 2} − *x*, {1\over 2} − *y*, −*z*

*Ow1 indicates the oxygen atom of the water mol­ecule with an occupancy of 0.224 (5).

**Table 3:** Percentage contributions of inter­atomic contacts to the Hirshfeld surface for the title compound

Contact	Percentage contribution
O⋯H/H⋯O	41.2
H⋯H	19.2
C⋯H/H⋯C	12.2
C⋯O/O⋯C	8.4
O⋯O	5.6
N⋯O/O⋯N	4.7
C⋯N/N⋯C	3.2
C⋯C	2.8
N⋯H/H⋯N	2.7

**Table 4:** Experimental details

Crystal data	
Chemical formula	C_14_H_12_N_4_O_7_·0.224H_2_O
*M*_r_	351.86
Crystal system, space group	Monoclinic, *C*2/*c*
Temperature (K)	150
*a*, *b*, *c* (Å)	24.2097 (11), 12.6311 (6), 10.4022 (5)
β (°)	113.133 (2)
*V* (Å^3^)	2925.2 (2)
*Z*	8
Radiation type	Mo *K*α
μ (mm^−1^)	0.13
Crystal size (mm)	0.34 × 0.32 × 0.27

Data collection	
Diffractometer	Bruker APEXII CCD
No. of measured, independent and observed [*I* > 2σ(*I*)] reflections	34832, 2944, 2699
*R*_int_	0.017
(sin θ/λ)_max_ (Å^−1^)	0.625

Refinement	
*R*[*F*^2^ > 2σ(*F* ^2^)], *wR*(*F* ^2^), *S*	0.034, 0.102, 1.04
No. of reflections	2944
No. of parameters	241
No. of restraints	6
H-atom treatment	H atoms treated by a mixture of independent and constrained refinement
Δρ_max_, Δρ_min_ (e Å^−3^)	0.29, −0.21

Computer programs: *APEX2* and *SAINT* (Bruker, 2007 ▸), *SHELXT* (Sheldrick, 2015*a* ▸), *SHELXL* (Sheldrick, 2015*b* ▸), *ORTEP-3 for Windows* (Farrugia, 2012 ▸) and *PLATON* (Spek, 2020 ▸).

## supplementary crystallographic information

### Crystal data

**Table:**

C_14_H_12_N_4_O_7_·0.224H_2_O	*F*(000) = 1454
*M**_r_* = 351.86	*D*_x_ = 1.598 Mg m^−^^3^
Monoclinic, *C*2/*c*	Mo *K*α radiation, λ = 0.71073 Å
*a* = 24.2097 (11) Å	Cell parameters from 9840 reflections
*b* = 12.6311 (6) Å	θ = 3.2–26.4°
*c* = 10.4022 (5) Å	µ = 0.13 mm^−^^1^
β = 113.133 (2)°	*T* = 150 K
*V* = 2925.2 (2) Å^3^	Block, orange
*Z* = 8	0.34 × 0.32 × 0.27 mm

### Data collection

**Table:**

Bruker APEXII CCD diffractometer	*R*_int_ = 0.017
φ and ω scans	θ_max_ = 26.4°, θ_min_ = 3.2°
34832 measured reflections	*h* = −30→30
2944 independent reflections	*k* = −15→15
2699 reflections with *I* > 2σ(*I*)	*l* = −13→13

### Refinement

**Table:**

Refinement on *F*^2^	6 restraints
Least-squares matrix: full	Hydrogen site location: mixed
*R*[*F*^2^ > 2σ(*F*^2^)] = 0.034	H atoms treated by a mixture of independent and constrained refinement
*wR*(*F*^2^) = 0.102	*w* = 1/[σ^2^(*F*_o_^2^) + (0.0586*P*)^2^ + 2.1077*P*] where *P* = (*F*_o_^2^ + 2*F*_c_^2^)/3
*S* = 1.04	(Δ/σ)_max_ < 0.001
2944 reflections	Δρ_max_ = 0.29 e Å^−^^3^
241 parameters	Δρ_min_ = −0.21 e Å^−^^3^

### Special details

**Table:**

Geometry. All esds (except the esd in the dihedral angle between two l.s. planes) are estimated using the full covariance matrix. The cell esds are taken into account individually in the estimation of esds in distances, angles and torsion angles; correlations between esds in cell parameters are only used when they are defined by crystal symmetry. An approximate (isotropic) treatment of cell esds is used for estimating esds involving l.s. planes.

### Fractional atomic coordinates and isotropic or equivalent isotropic displacement parameters (Å^2^)

**Table:**

	*x*	*y*	*z*	*U*_iso_*/*U*_eq_	Occ. (<1)
C1	0.37057 (5)	0.39423 (9)	0.82909 (12)	0.0190 (2)	
C2	0.37833 (5)	0.39902 (9)	0.97648 (12)	0.0207 (2)	
C3	0.48693 (5)	0.37742 (9)	1.04270 (12)	0.0208 (2)	
C4	0.42279 (5)	0.38365 (8)	0.79361 (12)	0.0186 (2)	
C5	0.24165 (5)	0.38344 (8)	0.50833 (12)	0.0192 (2)	
C6	0.23130 (5)	0.37794 (9)	0.36716 (12)	0.0204 (2)	
H6	0.263906	0.380695	0.338172	0.025*	
C7	0.17254 (5)	0.36834 (9)	0.26874 (11)	0.0197 (2)	
C8	0.12459 (5)	0.36539 (9)	0.31079 (12)	0.0201 (2)	
H8	0.084612	0.358679	0.243355	0.024*	
C9	0.13569 (5)	0.37237 (9)	0.45277 (12)	0.0200 (2)	
C10	0.19423 (5)	0.38111 (9)	0.55282 (12)	0.0203 (2)	
H10	0.201704	0.385399	0.649417	0.024*	
C11	0.15859 (5)	0.35937 (9)	0.11562 (12)	0.0211 (2)	
C12	0.19700 (5)	0.35182 (11)	−0.05977 (12)	0.0264 (3)	
H12A	0.177183	0.284238	−0.096410	0.040*	
H12B	0.235389	0.355077	−0.070599	0.040*	
H12C	0.171209	0.410245	−0.111573	0.040*	
C13	0.08236 (5)	0.37078 (10)	0.49220 (12)	0.0231 (3)	
C14	0.04671 (6)	0.39831 (12)	0.66992 (14)	0.0307 (3)	
H14A	0.022390	0.461429	0.630597	0.046*	
H14B	0.062181	0.401216	0.772194	0.046*	
H14C	0.021864	0.334851	0.636695	0.046*	
N1	0.43716 (4)	0.38898 (8)	1.07229 (10)	0.0215 (2)	
H1N	0.445330	0.388946	1.160377	0.026 (4)*	
N2	0.47752 (4)	0.37483 (8)	0.90340 (10)	0.0209 (2)	
H2N	0.510968	0.367522	0.886519	0.032 (4)*	
N3	0.31416 (4)	0.39456 (7)	0.73771 (10)	0.0197 (2)	
N4	0.30173 (4)	0.38952 (8)	0.60489 (10)	0.0202 (2)	
H4N	0.329280	0.391432	0.572748	0.041 (5)*	
O1	0.33809 (4)	0.41106 (8)	1.01707 (9)	0.0285 (2)	
O2	0.53768 (4)	0.37191 (8)	1.13392 (9)	0.0279 (2)	
O3	0.41946 (4)	0.38163 (7)	0.67172 (8)	0.0229 (2)	
O4	0.10827 (4)	0.35077 (8)	0.02776 (9)	0.0310 (2)	
O5	0.20773 (4)	0.36060 (7)	0.08791 (8)	0.0250 (2)	
O6	0.03258 (4)	0.35068 (10)	0.41153 (10)	0.0431 (3)	
O7	0.09668 (4)	0.39450 (8)	0.62583 (9)	0.0292 (2)	
OW1	0.3463 (2)	0.3484 (4)	0.3151 (6)	0.0466 (19)	0.224 (5)

### Atomic displacement parameters (Å^2^)

**Table:**

	*U*^11^	*U*^22^	*U*^33^	*U*^12^	*U*^13^	*U*^23^
C1	0.0158 (5)	0.0212 (5)	0.0187 (5)	−0.0008 (4)	0.0054 (4)	−0.0001 (4)
C2	0.0184 (5)	0.0233 (6)	0.0203 (6)	−0.0022 (4)	0.0074 (5)	−0.0026 (4)
C3	0.0192 (5)	0.0237 (6)	0.0179 (5)	0.0009 (4)	0.0054 (4)	−0.0003 (4)
C4	0.0166 (5)	0.0198 (5)	0.0175 (5)	−0.0002 (4)	0.0047 (4)	0.0010 (4)
C5	0.0157 (5)	0.0195 (5)	0.0193 (5)	−0.0003 (4)	0.0034 (4)	0.0016 (4)
C6	0.0188 (5)	0.0220 (5)	0.0209 (6)	0.0006 (4)	0.0081 (4)	0.0018 (4)
C7	0.0203 (5)	0.0198 (5)	0.0177 (6)	0.0005 (4)	0.0061 (4)	0.0010 (4)
C8	0.0171 (5)	0.0211 (5)	0.0189 (5)	−0.0002 (4)	0.0034 (4)	0.0003 (4)
C9	0.0173 (5)	0.0223 (5)	0.0193 (5)	−0.0007 (4)	0.0061 (4)	0.0006 (4)
C10	0.0197 (5)	0.0229 (5)	0.0172 (5)	−0.0006 (4)	0.0061 (4)	0.0006 (4)
C11	0.0210 (5)	0.0222 (5)	0.0192 (5)	0.0008 (4)	0.0070 (4)	0.0011 (4)
C12	0.0253 (6)	0.0365 (7)	0.0180 (6)	0.0003 (5)	0.0092 (5)	0.0008 (5)
C13	0.0185 (6)	0.0295 (6)	0.0192 (5)	−0.0006 (4)	0.0053 (4)	0.0002 (4)
C14	0.0221 (6)	0.0462 (8)	0.0262 (6)	−0.0023 (5)	0.0121 (5)	−0.0036 (5)
N1	0.0184 (5)	0.0310 (5)	0.0141 (5)	−0.0001 (4)	0.0054 (4)	−0.0023 (4)
N2	0.0149 (5)	0.0304 (5)	0.0167 (5)	0.0025 (4)	0.0056 (4)	0.0005 (4)
N3	0.0174 (5)	0.0219 (5)	0.0181 (5)	−0.0007 (3)	0.0053 (4)	−0.0001 (3)
N4	0.0153 (5)	0.0270 (5)	0.0176 (5)	−0.0005 (3)	0.0055 (4)	0.0017 (4)
O1	0.0192 (4)	0.0439 (5)	0.0244 (4)	−0.0026 (4)	0.0108 (3)	−0.0066 (4)
O2	0.0190 (4)	0.0441 (5)	0.0169 (4)	0.0043 (4)	0.0032 (3)	−0.0004 (3)
O3	0.0178 (4)	0.0344 (5)	0.0161 (4)	−0.0001 (3)	0.0062 (3)	0.0014 (3)
O4	0.0211 (4)	0.0510 (6)	0.0187 (4)	−0.0024 (4)	0.0056 (3)	−0.0028 (4)
O5	0.0204 (4)	0.0370 (5)	0.0172 (4)	0.0008 (3)	0.0071 (3)	0.0015 (3)
O6	0.0175 (4)	0.0853 (8)	0.0252 (5)	−0.0085 (5)	0.0069 (4)	−0.0130 (5)
O7	0.0190 (4)	0.0498 (6)	0.0193 (4)	−0.0038 (4)	0.0080 (3)	−0.0038 (4)
OW1	0.031 (3)	0.056 (3)	0.058 (3)	0.001 (2)	0.024 (2)	0.004 (2)

### Geometric parameters (Å, º)

**Table:**

C1—N3	1.3223 (15)	C9—C10	1.3945 (16)
C1—C4	1.4557 (16)	C9—C13	1.5006 (16)
C1—C2	1.4708 (15)	C10—H10	0.9500
C2—O1	1.2140 (14)	C11—O4	1.2068 (14)
C2—N1	1.3864 (14)	C11—O5	1.3298 (14)
C3—O2	1.2242 (14)	C12—O5	1.4581 (14)
C3—N1	1.3638 (15)	C12—H12A	0.9800
C3—N2	1.3759 (15)	C12—H12B	0.9800
C4—O3	1.2387 (14)	C12—H12C	0.9800
C4—N2	1.3724 (14)	C13—O6	1.1944 (15)
C5—C6	1.3909 (16)	C13—O7	1.3280 (15)
C5—C10	1.3968 (16)	C14—O7	1.4533 (14)
C5—N4	1.4080 (14)	C14—H14A	0.9800
C6—C7	1.3936 (16)	C14—H14B	0.9800
C6—H6	0.9500	C14—H14C	0.9800
C7—C8	1.3926 (16)	N1—H1N	0.8580
C7—C11	1.4970 (15)	N2—H2N	0.8982
C8—C9	1.3963 (16)	N3—N4	1.2950 (14)
C8—H8	0.9500	N4—H4N	0.8557
			
N3—C1—C4	124.93 (10)	O4—C11—O5	124.04 (10)
N3—C1—C2	114.97 (10)	O4—C11—C7	123.45 (10)
C4—C1—C2	120.00 (10)	O5—C11—C7	112.50 (9)
O1—C2—N1	119.97 (10)	O5—C12—H12A	109.5
O1—C2—C1	125.19 (10)	O5—C12—H12B	109.5
N1—C2—C1	114.84 (9)	H12A—C12—H12B	109.5
O2—C3—N1	122.53 (10)	O5—C12—H12C	109.5
O2—C3—N2	121.04 (10)	H12A—C12—H12C	109.5
N1—C3—N2	116.41 (10)	H12B—C12—H12C	109.5
O3—C4—N2	120.22 (10)	O6—C13—O7	124.04 (11)
O3—C4—C1	123.21 (10)	O6—C13—C9	123.36 (11)
N2—C4—C1	116.56 (10)	O7—C13—C9	112.60 (9)
C6—C5—C10	121.20 (10)	O7—C14—H14A	109.5
C6—C5—N4	117.59 (10)	O7—C14—H14B	109.5
C10—C5—N4	121.20 (10)	H14A—C14—H14B	109.5
C5—C6—C7	119.25 (10)	O7—C14—H14C	109.5
C5—C6—H6	120.4	H14A—C14—H14C	109.5
C7—C6—H6	120.4	H14B—C14—H14C	109.5
C8—C7—C6	120.52 (10)	C3—N1—C2	126.63 (9)
C8—C7—C11	117.69 (10)	C3—N1—H1N	112.8
C6—C7—C11	121.79 (10)	C2—N1—H1N	120.6
C7—C8—C9	119.52 (10)	C4—N2—C3	125.51 (10)
C7—C8—H8	120.2	C4—N2—H2N	119.7
C9—C8—H8	120.2	C3—N2—H2N	114.8
C10—C9—C8	120.75 (11)	N4—N3—C1	120.55 (10)
C10—C9—C13	121.89 (10)	N3—N4—C5	120.38 (9)
C8—C9—C13	117.35 (10)	N3—N4—H4N	121.7
C9—C10—C5	118.75 (10)	C5—N4—H4N	117.9
C9—C10—H10	120.6	C11—O5—C12	115.04 (9)
C5—C10—H10	120.6	C13—O7—C14	115.50 (9)
			
N3—C1—C2—O1	−5.85 (17)	C6—C7—C11—O5	0.66 (15)
C4—C1—C2—O1	177.58 (11)	C10—C9—C13—O6	−170.65 (13)
N3—C1—C2—N1	174.41 (9)	C8—C9—C13—O6	9.75 (18)
C4—C1—C2—N1	−2.17 (15)	C10—C9—C13—O7	9.60 (16)
N3—C1—C4—O3	5.51 (18)	C8—C9—C13—O7	−170.00 (10)
C2—C1—C4—O3	−178.27 (10)	O2—C3—N1—C2	177.97 (11)
N3—C1—C4—N2	−173.94 (10)	N2—C3—N1—C2	−0.46 (17)
C2—C1—C4—N2	2.27 (15)	O1—C2—N1—C3	−178.47 (11)
C10—C5—C6—C7	−0.97 (16)	C1—C2—N1—C3	1.29 (17)
N4—C5—C6—C7	177.91 (10)	O3—C4—N2—C3	179.07 (11)
C5—C6—C7—C8	0.67 (16)	C1—C4—N2—C3	−1.46 (16)
C5—C6—C7—C11	−178.47 (10)	O2—C3—N2—C4	−177.92 (11)
C6—C7—C8—C9	0.14 (16)	N1—C3—N2—C4	0.53 (17)
C11—C7—C8—C9	179.32 (10)	C4—C1—N3—N4	−2.91 (17)
C7—C8—C9—C10	−0.68 (16)	C2—C1—N3—N4	−179.30 (9)
C7—C8—C9—C13	178.92 (10)	C1—N3—N4—C5	176.15 (10)
C8—C9—C10—C5	0.40 (16)	C6—C5—N4—N3	−179.84 (10)
C13—C9—C10—C5	−179.18 (10)	C10—C5—N4—N3	−0.97 (16)
C6—C5—C10—C9	0.43 (16)	O4—C11—O5—C12	0.65 (16)
N4—C5—C10—C9	−178.40 (10)	C7—C11—O5—C12	179.85 (9)
C8—C7—C11—O4	0.70 (17)	O6—C13—O7—C14	−1.96 (19)
C6—C7—C11—O4	179.87 (11)	C9—C13—O7—C14	177.79 (10)
C8—C7—C11—O5	−178.51 (10)		

### Hydrogen-bond geometry (Å, º)

*Cg*2 is the centroid of the C5–C10 benzene ring.

**Table:**

*D*—H···*A*	*D*—H	H···*A*	*D*···*A*	*D*—H···*A*
N1—H1*N*···O2^i^	0.86	2.03	2.8800 (13)	174
N2—H2*N*···O3^ii^	0.90	2.01	2.8931 (15)	168
N4—H4*N*···O3	0.86	2.02	2.6571 (15)	131
N4—H4*N*···O1^iii^	0.86	2.59	2.9302 (14)	105
C6—H6···Ow1	0.95	2.14	3.061 (6)	163
C12—H12*B*···O1^iv^	0.98	2.39	3.2743 (17)	149
C14—H14*B*···O4^v^	0.98	2.53	3.4754 (16)	163
C12—H12*C*···*Cg*2^iii^	0.98	2.73	3.4717 (15)	133

Symmetry codes: (i) −*x*+1, *y*, −*z*+5/2; (ii) −*x*+1, *y*, −*z*+3/2; (iii) *x*, −*y*+1, *z*−1/2; (iv) *x*, *y*, *z*−1; (v) *x*, *y*, *z*+1.
